# Association between ANGPTL-4 and the proinflammatory process in cancer cachexia patients

**DOI:** 10.18632/oncotarget.27269

**Published:** 2019-11-05

**Authors:** Nelson Inácio Pinto Neto, Valter Tadeu Boldarine, Ana Claudia Losinskas Hachul, Lila Missae Oyama, Joanna Darck Carola Correia Lima, Estefania Simoes Fernandez, José Pinhata Otoch, Paulo Sérgio Martins de Alcântara, Flavio Tokeshi, Marilia Cerqueira Leite Seelaender, Claudia Maria da Penha Oller do Nascimento

**Affiliations:** ^1^ Departamento de Fisiologia, Universidade Federal de São Paulo, Escola Paulista de Medicina, São Paulo, Brazil; ^2^ Department of Clinical Surgery, University of São Paulo, São Paulo, Brazil; ^3^ Institute of Biomedical Sciences, Cancer Metabolism Research Group, University of São Paulo, São Paulo, Brazil

**Keywords:** ANGPTL-4, cytokines, tumor, cachexia, proinflammatory process

## Abstract

**Background:**

Contradictory results are reported for the role of angiopoietin-like 4 (ANGPTL-4) in the development of cancer-cachexia and inflammation, given its importance in angiogenesis and inflammatory signaling. Our aim was to analyze the levels of ANGPTL-4 in colorectal cancer patients with a stable weight and those with cachexia in order to establish a relationship between ANGPTL-4 and the inflammatory process.

**Results:**

Plasma and tumor levels of ANGPTL-4 were higher in CC in comparison to other groups. A positive association was verified between plasmatic ANGPTL-4 and NFκB levels in tumor from CC. In WSC, we identified an association between the plasmatic ANGPTL-4, IL-15, and IL-10 in tumor and IL-15 in MES. Increased levels of NFκB and TNF-R1 in MES were detected in CC in comparison to WSC. Specifically in CC-group, a positive correlation was found between ANGPTL-4 levels and those of IL-1β, TNF-α, and NFκB in tumor, along with an association between ANGPTL-4 levels with IL-1β and MCP-1 levels in tumor; and ANGPTL-4 and IL-1β levels in MES.

**Methods:**

We studied 102 patients, who were divided into three groups: control patients (C, n=37), cancer patients with a stable weight (WSC, n=23), and cancer-cachexia patients (CC, n=42). Samples of plasma, tumor, mesenteric (MES) and subcutaneous adipose tissue were removed for the determination of ANGPTL-4 levels and other proinflammatory factors.

**Conclusions:**

ANGPTL-4 levels were higher in plasma and tumor of CC-group, and positively associated with pro-inflammatory and pro-tumorigenic factors. Our results suggest an opposite effect of ANGPTL-4 depending on the concentration and presence of cachexia.

## INTRODUCTION

Cancer cachexia is a systemic disease affecting the vast majority of patients with end stage cancer, contributing to poor quality of life and decreased life expectancy [1, 2]. Cachexia is characterized by an involuntary loss of skeletal muscle and adiposity, decreasing functional quality of life.

In cancer-cachexia, adipose tissue suffers excessive lipolysis, decreased lipogenesis *de novo* and uptake of fatty acids from triacylglycerol from lipoproteins by a reduction on activity of lipoprotein lipase [3]. All these metabolic alterations contribute to adipose tissue wasting. Also, it has been reported that, in cancer cachexia, white adipose cells suffering ‘browning’ increase heat production and energy expenditure. Recently, it has been described a participation of Src family kinase as a negative regulator of browning [4].

This disease is a multifactorial syndrome with a complex etiology that influences the dysfunction of several metabolic pathways. Cachexia´s etiology correlates to negative protein and energy balance, as a consequence of impaired metabolism and reduced food intake; traditional diet therapy is not sufficient for improvement [5, 6].

The aggressiveness of a tumor often depends of the development of metastatic lesions, which are characterized by the proliferation of cancer cells, and cancer associated cachexia, related to products derived from the tumor microenvironment or the secretion of proinflammatory factors by other tissues [7].

C-reactive protein is part of the IL-1β, IL-6 inflammatory cascade, and may serve as a marker of systemic low-grade inflammation [8]. Systemic inflammation is a hallmark of cancer patients, with the inflammatory process being one of the factors responsible for the metabolic changes characteristic of cancer [9].

Evidence indicates that other cell types present in the tumor microenvironment also play key roles in the development of the cancer. Inflamed adipose tissue can also modify the tumor microenvironment, which likely explains the relationship between adiposity and several tumors [10].

There are multiple sources of such inflammatory factors: tumor cells, activated immune cells, adipocytes and myocytes, which promote the release of tumorkines, adipokines, myokines, and other inflammatory mediators such as IL-6, TNF-α, IL-1, NFҡB, MCP-1, and other factors involved in tumor growth, angiogenesis and apoptosis process as Fas-associated death domain protein (FADD) [1, 11]. Also, the downregulation of the anti-inflammatory factors as IL-10 and IL-15 contribute for the development of the tumor and loss of body mass [12].

One of the proteins involved in cancer cachexia syndrome is angiopoietin-like 4 (ANGPTL-4), which has been classified as an adipokine due to its high expression in adipose tissues and liver. Also, it was reported that ANGPTL-4 is expressed in a variety of tissues (guts, liver, adipose tissues and others) as well as tumors [7]. Furthermore, ANGPTL-4 is involved in vascular permeability, angiogenesis, and inflammatory signaling, and may also be involved in the progression of metastatic tumors [13].

Certain authors suggested that ANGPTL-4 prevents tumor metastasis by inhibiting vascular permeability, tumor cell motility, angiogenesis, and invasiveness; thus, ANGPTL-4 may have an antitumor effect, decreasing tumor growth and metastasis [14, 15]. Conversely, ANGPTL-4 mRNA expression was reported to be increased in the perinecrotic areas of human tumors [16, 17]. In addition, the expression of ANGPTL-4 is positively correlated to the increasing malignity of tumors, suggesting a role of ANGPTL-4 in tumor growth [18].

Hypoxia, a phenomenon highly frequent in malignant solids tumors, is linked to an increased gene and protein expression and secretion of ANGPTL-4, causing chronic inflammation and macrophage infiltration; therefore, it is reasonable to suggest that ANGPTL-4 is largely connected to inflammation. Toll-like receptor 4 (TLR-4) activators are believed to regulate ANGPTL-4, and the expression of ANGPTL-4 may also be regulated by NFҡB [19]. Furthermore, several cytokines, such as interleukins, TNF-α, and IFN-γ have been highlighted as being involved in ANGPTL-4 expression.

Since understanding the association between ANGPTL-4 and inflammatory factors may suggest an important therapeutic target in the cancer cachexia, and the existence of an important cross talk between adipose tissue and tumor, the present study aimed to analyze ANGPTL-4 levels in plasma, tumor and adipose tissue from colorectal cancer patients with a stable weight and those with cachexia and evaluate the association of ANGPTL-4 in the inflammatory process.

## RESULTS

### General subject characteristics

Table 1 depicts patients’ general characteristics. The CC group showed a low serum hemoglobin concentration and significant weight loss compared to the other two groups. The BMI and serum albumin concentration were lower in the CC group in comparison to the C group. The CRP and CRP/albumin ratio were higher in the CC and WSC groups than in the C group. Also, as determined by the EORTC-QLQ-C30 all the cancer cachectic patient presented anorexia and fatigue.

**Table 1:** General characteristics of patients

	C	WSC	CC
**n**	37	23	42
**Gender**	F (11) / M ( 26)	F (7) / M ( 16)	F (16) / M ( 26)
**Age (years)**	53.19 ± 2.17	60.57 ± 3.05	62.33 ± 2.05 ^*^
**BMI (kg/m2)**	25.97 ± 0.48	24.52 ± 0.69	23.06 ± 0.55 ^*^
**Weight loss (kg)**	0.0 [ 0.0 - 1.30]	0.0 [ 0.0 - 5.45]	8.450 [2.00 - 30.50] ^*#^
**Weight loss (%)**	0.0 [ 0.0 - 1.88]	0.0 [0. 4.90]	12.04 [3.96 - 32.93] ^*#^
**CRP (mg/L )**	1.30 [ 0.10 - 7.30]	4.60 [0.20 - 16.90]^*^	10.80 [0.20 - 16.80]^*^
**ALBUMIN (g/dL)**	4.58 [3.22 - 6.81]	4.31 [2.24 - 6.00]	4.06 [0.84 - 5.41]^*^
**CRP/ ALBUMIN**	0.03 [0.00 - 0.16]	0.11 [0.00- 0.75]^*^	0.27 [0.00 - 1.58]^*^
**Hemoglobin (g/dL)**	15.09 ± 0.25	14.13 ± 0.47	11.10 ± 0.33 ^*#^
**Tumor stage (n)**			
**I-II**		13	15
**III- IV**		10	27

Control patient (C) weight-stable cancer patient (WSC) and in cachectic cancer patient (CC). Data expressed as median [minimum; maximum] or as mean ± SEM. Number of patient are showed in parenthesis. ^*^ p<0.05 in relation to C; ^#^ p<0.05 in relation to WSC.

### Protein expression analysis

**Figure 1:**
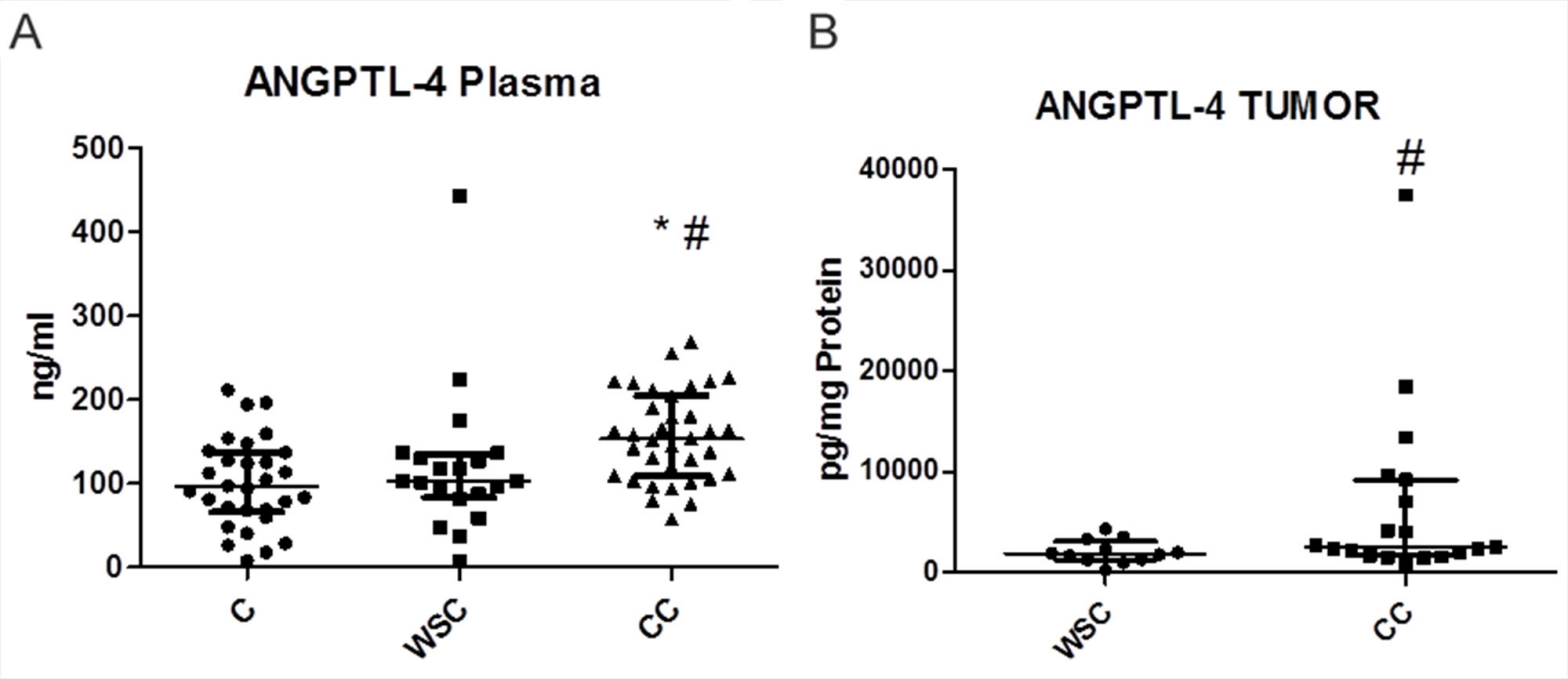
Dot plot (median wich interquartile range) representing plasma (ng/mL- A) and tumor (pg/mg of protein- B) ANGPTL-4 concentration in each patient Control (C), weight-stable cancer patient (WSC) and in cachectic cancer patient (CC). ^*^ p<0.05 in relation to C; ^#^ p<0.05 in relation to WSC.

The concentrations of ANGPTL-4 in plasma (Figure 1A) and tumor tissue (Figure 1B) were higher in the CC group. The concentration of ANGPTL-4 was not modified by the tumor stage in WSC group (p= 0.89), CC group (0.07) and between I-II and III-IV tumor stage (p=0.09).

In the mesenteric and subcutaneous adipose tissue, significant differences in ANGPTL-4 levels were not detected among the groups (Table 2). Table 3 shows the concentrations of various proinflammatory cytokines/chemokines, tumor growth factors, signaling proteins involved in proinflammatory pathways, and cachexia related factors in tumor, mesenteric and subcutaneous adipose tissues. There were no significant differences among the groups with respect to the majority of proteins analyzed; nevertheless, NFκB (p < 0.0582) and FADD (p < 0.01) levels were significantly lower in the tumor tissue in the CC group than that in the WSC group.

**Table 2:** Concentration of ANGPTL-4 in control patient, weight-stable cancer patient (WSC) and in cachectic cancer patient (CC). Data expressed as median [minimum; maximum] or as mean ± SEM. n = 6–36 per group

	C	WSC	CC	p
**Plasma (ng/ml)**	95.87[8.03– 211.60]	102.80 [7.82 – 442.80]	153.40 [57.76 – 268.90]^*#^	0.0004
**Tumor (pg/mg ptn)**		1851.00 [290.10 – 4342.00]	2542.00 [929.20-3752.80]^#^	0.05
**Mesenteric adipose tissue (pg/mg ptn)**	10779.00 [5855.00 – 20824.00]	8816.00 [3535.00 -26085.00]	11292.00 [6079.00-35970.00]	0.47
**Subcutaneous adipose tissue (pg/mg ptn)**	34350.00 [14872.00 -99796.00]	25548.00 [13553.00 -52286.00]	34748.00 [21836.00 -59668.00]	0.41

^*^p<0.05 in relation to C;

^#^p<0.05 in relation to WSC.

**Table 3:** Tumor, mesenteric white adipose tissue and subcutaneous white adipose tissue growth factors and protein content (pg/ mg of protein or MFI - Medium fluorescent intensity) in weight-stable cancer patient (WSC) and in cachectic cancer patient (CC). Data expressed as median [minimum; maximum] or as mean ± SEM. n = 5–11 per group

	Tumor			Mesenteric adipose tissue			Subcutaneous adipose tissue			
	WSC	CC	p	WSC	CC	p	C	WSC	CC	p
**NFkB**	55.00[42.00 -87.00]	47.50[44.00- 53.00]	0.0582	70.00 [38.00 – 82.00]	93.00 [81.00-142.50]^#^	0.01	74.00[54.00-180.00]	96.00[63.00-166.00]	91.00[68.00-126.50]	0.15
**TNFR-1**	133.90±17.74	95.71±14.35	0.11	146.00 [117.50-197.00]	241.00[167.00-287.00]^#^	0.03	269.50±44.64	221.70±21.13	223.70±15.95	0.44
**c-MYC**	178.70±33.63	111.70±12.55	0.10	111.00[98.00-138.00]	153.50[103.00-200.00	0.07	99.75±6.28	114.80±8.88	120.50±6.30	0.11
**FADD**	264.40±34.56	136.60±31.45^#^	0.01	133.00[108.00-398.00]	181.00[104.50-289.50]	0.84	169.90±27.40	162.80±16.09	176.80±22.68	0.91
**IKK**	56.00[50.00-76.00]	54.00[48.00-61.00]	0.36	76.00[50.00 -91.00]	94.00[61.00-158.00]	0.18	75.80±5.68	78.95±4.69	74.65±3.33	0.78
**IkB**	102.00[52.00-362.00]	86.00[57.00-373.00]	0.95	222.50[128.00-365.00]	334.50[125.00-498.00]	0.54	174.50[115.00-925.50]	316.50[159.00-1466.00]	224.00[164.00-285.00]	0.13
**Src**	61.89±3.08	64.14±3.29	0.60	67.00[63.00-93.00]	61.00[57.00-71.00]	0.12	56.64±3.41	57.50±3.09	64.85±2.98	0.13
**ERK-MAPK 1/2**	560.60±118.10	555.50±84.28	0.87	908.50[244.00-1938.00]	1025.00[428.00-1710.00]	1.00	1117.00±231.05	803.90±177.02	1394.00±314.57	0.26
**STAT-1**	117.00[63.00-747.00]	86.50[68.00-97.00]	0.18	1471.00[546.00-1913.00]	1798.00[102.00-5315.00]	0.43	2097.00±349.11	2159.00±331.30	2661.00±669.93	0.83
**STAT-3**	69.38±3.76	70.86±3.18	0.76	126.00[104.99-135.00]	113.50[68.00-143.50]	0.79	150.00[62.50-798.00]	184.00[74.00-472.00]	115.00[72.00-310.00]	0.80

^#^p<0.05 in relation to WSC.

The mesenteric adipose tissue in the CC group presented a significantly higher level of NFκB (p< 0.01) and TNFR-1 (p = 0.03) than that in the WSC group. The c-MYC concentration, although not statistically different, showed a tendency to be higher in the mesenteric adipose tissue in the CC group (p = 0.07).

It is important to highlight that the mesenteric adipose tissue samples of patients in the C group were difficult to obtain due to the surgical procedure; thus, it was only possible to evaluate the levels of the factors in the C group in the subcutaneous adipose tissue. The levels of all the factors analyzed in this tissue were similar among the three groups.

### Correlation and association analysis

**Figure 2:**
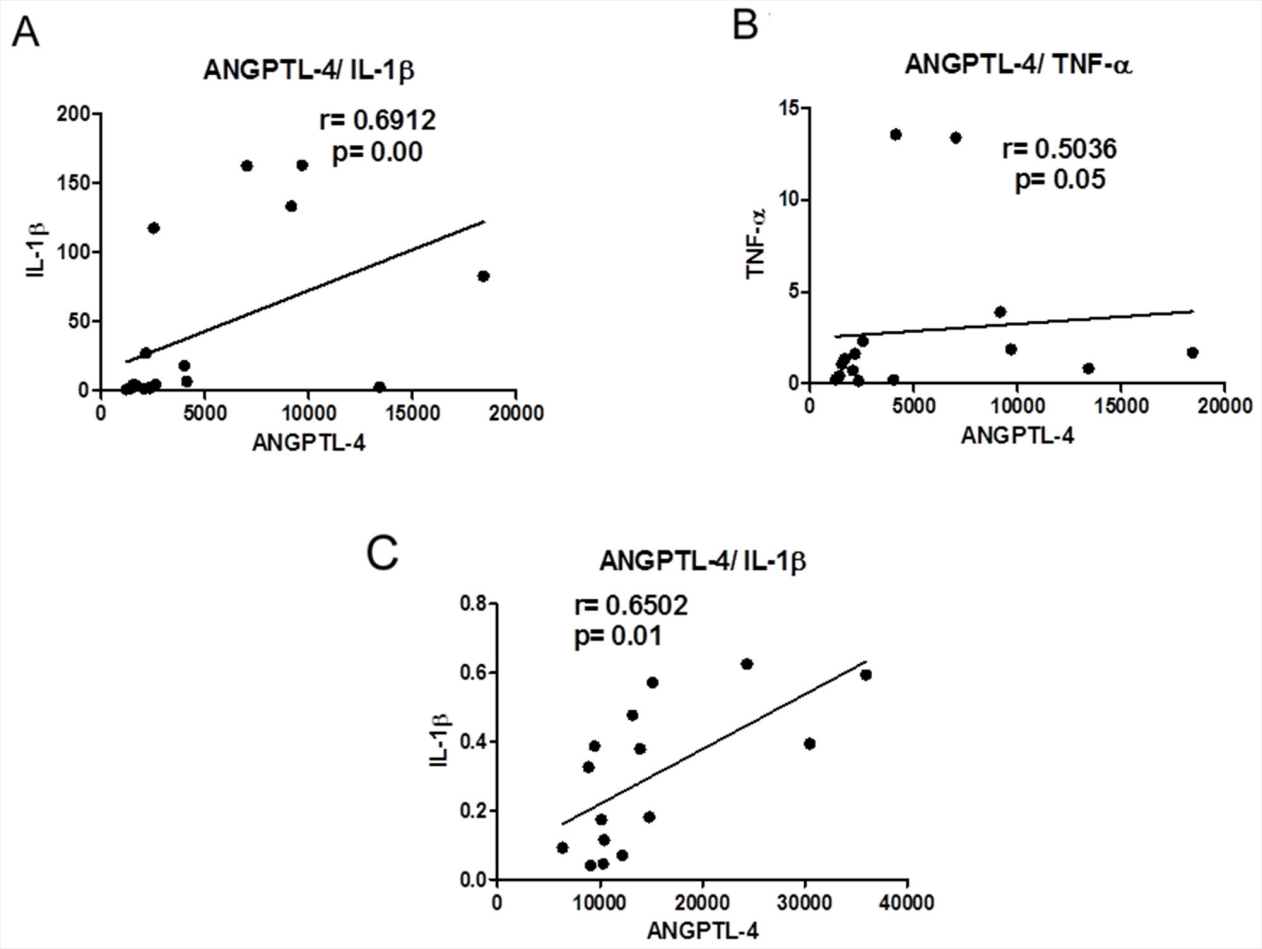
**(A)** Correlation between ANGPTL-4 and IL-1β in tumor of cancer with cachexia patients. **(B)** Correlation between ANGPTL-4 and TNF-α in tumor of cancer with cachexia patients. **(C)** Correlation between ANGPTL-4 and IL-1β in mesenteric adipose tissue of cancer with cachexia patients.

Correlation analysis (Spearman or Pearson, for non-parametric and parametric data, respectively, as stated in Tables 4 and 5 and Figure 2A–2C) aimed to elucidate the relationships between the levels of cytokines/chemokines and ANGPTL-4 in the tumor, mesenteric and subcutaneous adipose tissues. In the WSC group, ANGPTL-4 showed a negative correlation with IL-10 (p < 0.05) in mesenteric adipose tissue. In tumor tissue in the CC group, ANGPTL-4 showed a significant positive correlation with IL-1β (p < 0.01; Figure 2A), a positive correlation with TNF-α (p ≤ 0.05; Figure 2B), and a positive correlation with NFκB (p < 0.05). The correlation between ANGPTL-4 and MCP-1 was not significantly different but showed a positive tendency (p = 0.06). A positive correlation between ANGPTL-4 and IL-1β was observed in the CC group in the mesenteric adipose tissue but not in the WSC group (Figure 2C). Subcutaneous adipose tissue showed no significant correlations.

**Table 4:** Correlation between protein expression of ANGPTL-4 and inflammatory factors in tumor, mesenteric white adipose tissue and subcutaneous white adipose tissue from WSC group and CC group. n = 5- 10

WSC group						
	Tumor		Mesenteric adipose tissue		Subcutaneous adipose tissue	
	r	P	r	p	r	p
**IL-1β**	0.1758	0.63	0.5357	0.23	-0.3714	0.49
**IL-6**	-0.1667	0.70	0.3571	0.44	-0.1429	0.80
**IL-10**	0.0385	0.92	-0.7133	0.01	-0.3571	0.44
**1L-15**	-0.2200	0.57	0.0357	0.96	-0.7143	0.09
**MCP-1**	-0.0424	0.92	0.3214	0.50	-0.6000	0.24
**TNF-α**	-0.5714	0.20	0.3412	0.50	-0.2143	0.66
**VEGF**	-0.1000	0.81	-0.8000	0.33	-0.4643	0.30
**CC group**						
	**Tumor**		**Mesenteric adipose tissue**		**Subcutaneous adipose tissue**	
	**r**	**P**	**r**	**p**	**r**	**p**
**IL-1β**	0.6912	0.00	0.6502	0.01	-0.2970	0.41
**IL-6**	0.2857	0.34	-0.2588	0.33	0.4167	0.27
**IL-10**	0.2308	0.45	0.2242	0.54	0.1382	0.72
**1L-15**	0.3431	0.18	-0.1250	0.66	0.0833	0.84
**MCP-1**	0.4559	0.06	-0.1529	0.57	0.2606	0.47
**TNF-α**	0.5036	0.05	0.3964	0.14	0.2864	0.45
**VEGF**	0.1789	0.49	0.2393	0.39	0.1400	0.72

**Table 5:** Correlation between protein expression of ANGPTL-4 and factors in tumor and mesenteric adipose tissue from CC group and WSC group. n = 5- 8

Tumor					Mesenteric adipose tissue			
	WSC		CC		WSC		CC	
	r	p	r	p	r	p	r	p
**NFκB**	-0.1102	0.79	0.4322	0.04	-0.1000	0.95	-0.6000	0.24
**FADD**	-0.5034	0.20	-0.5714	0.20	-0.5000	0.45	-0.1996	0.70
**TNFR-1**	0.0010	0.99	-0.3929	0.39	0.0395	0.95	-0.4286	0.42
**c-MYC**	-0.3211	0.44	-0.6071	0.17	-0.3906	0.52	0.08571	0.92

In WSC group the plasma level of ANGPTL-4 was positively correlated with IL-10 (p=0.04) and IL-15 (p=0.02) in the tumor and with IL-15 (p < 0.01) in the mesenteric adipose tissue (Table 6). A positive correlation between plasma ANGPTL-4 and NFκB (p< 0.01) in the tumor was detected in CC group (Table 7).

**Table 6:** Correlation between plasma levels of ANGPTL-4 and inflammatory factors content in tumor, mesenteric white adipose tissue and subcutaneous white adipose tissue from WSC group and CC group. n = 5- 10

WSC group						
	Tumor		Mesenteric adipose tissue		Subcutaneous adipose tissue	
	r	P	r	p	r	p
**IL-1β**	-0.2857	0.34	-0.3571	0.39	-0.5429	0.30
**IL-6**	0.0060	0.99	0.5654	0.18	-0.1430	0.81
**IL-10**	0.6037	0.04	0.0489	0.89	-0.6000	0.24
**1L-15**	0.6880	0.02	0.9224	0.00	-0.2571	0.66
**MCP-1**	-0.0604	0.85	-0.0207	0.96	0.8000	0.13
**TNF-α**	0.3697	0.29	0.4813	0.27	-0.2571	0.66
**VEGF**	-0.3653	0.26	0.8000	0.75	-0.0857	0.92
**CC group**						
	**Tumor**		**Mesenteric adipose tissue**		**Subcutaneous adipose tissue**	
	**r**	**P**	**r**	**p**	**r**	**p**
**IL-1β**	0.0275	0.93	-0.3846	0.17	0.0714	0.91
**IL-6**	-0.1879	0.61	0.0065	0.99	0.2571	0.66
**IL-10**	0.2857	0.56	-0.1333	0.74	0.3714	0.50
**1L-15**	0.0010	0.99	-0.1538	0.62	0.7714	0.10
**MCP-1**	-0.0165	0.96	-0.2308	0.43	0.9429	0.02
**TNF-α**	-0.1879	0.61	-0.2857	0.34	0.3714	0.50
**VEGF**	0.0909	0.78	-0.3791	0.20	0.6000	0.35

**Table 7:** Correlation between plasma levels of ANGPTL-4 and expression factors in tumor and mesenteric adipose tissue from CC group and WSC group. n = 5- 8

Tumor					Mesenteric adipose tissue			
	WSC		CC		WSC		CC	
	r	p	r	p	r	p	r	p
**NFκB**	-0.1840	0.64	0.9076	0.00	0.3440	0.40	0.2500	0.59
**FADD**	0.2780	0.47	0.3651	0.42	0.3571	0.39	-0.0714	0.91
**TNFR-1**	0.2762	0.47	0.3571	0.44	-0.1429	0.75	-0.0614	0.90
**c-MYC**	0.4957	0.17	0.1947	0.67	0.2244	0.59	0.3884	0.39

Using the regression analysis we verified, in CC group, that ANGPL-4 content in the tumor was associated positively with IL-1β and MCP-1 contents; in the mesenteric adipose tissue with IL-1β content, and ANGPTL-4 plasma level with NFκB content in tumor (Table 8).

**Table 8:** Linear regression models for predictors of inflammatory parameters from CC group and WSC group. n = 6- 17

Predictors	Beta coefficients	Standard error	p
**Dependent variable:** Tumor IL-1β content on CC group			
ANGPTL-4	0.005886	0.002845	0.0563
**Dependent variable: **Tumor TNF-α content on CC group			
ANGPTL-4	0.00007855	0.0002349	0.7434
**Dependent variable: **Tumor MCP-1 content on CC group			
ANGPTL-4	0.03447	0.01182	0.0107
**Dependent variable: **Tumor NFκB content on CC group			
ANGPTL-4	-0.001530	0.002006	0.4799
**Dependent variable: **Mesenteric adipose tissue IL-1β content on CC group			
ANGPTL-4	0.00001594	0.000005164	0.0087
**Dependent variable: **Mesenteric adipose tissue IL-10 content on WSC group			
ANGPTL-4	-0.0001222	0.00004900	0.0318
**Dependent variable:** Tumor NFκB content on CC group			
ANGPTL-4 serum	0.2971	0.06147	0.0047
**Dependent variable:** Tumor IL-10 content on WSC group			
ANGPTL-4 serum	0.002416	0.001063	0.0492
**Dependent variable:** Tumor IL-15 content on WSC group			
ANGPTL-4 serum	0.01637	0.005754	0.0193
**Dependent variable:** Mesenteric adipose tissue IL-15 content on WSC group			
ANGPTL-4 serum	0.001445	0.0002706	0.0031

In the mesenteric adipose tissue of WSC group, a negative association was detected between ANGPTL-4 and IL-10 contents. Also, plasma ANGPTL-4 level was positively associated with IL-10 and IL-15 contents in tumor and with IL-15 content in mesenteric adipose tissue (Table 8).

## DISCUSSION

Our results indicate a more pronounced proinflammatory state in the mesenteric adipose tissue and tumor of colorectal cancer cachexia patients compared to those with a stable weight (Table 3). As of now, this seems to be the first study suggesting that ANGPTL-4 may play an important role in the modulation of colorectal cancer cachexia. An increase in plasma and tumor ANGPTL-4 was detected in cancer cachexia patient, as well as an association to proinflammatory factors such as NFκB, IL-1β and MCP-1. On the other hand, in weight stable patients, who had a low ANGPTL-4 levels in plasma and tumor, it was observed an association of this protein to IL-10 and IL-15, anti-inflammatory and anti-tumorigenic factor. Taken all together our results suggest that increased ANGPTL-4 levels may contribute for a proinflammatory environment on cancer cachexia patients.

The patients in the CC group had a significant weight loss over the previous 6 months, an increase in CRP levels, a decrease in serum hemoglobin concentration, and reported fatigue and/or anorexia. These conditions confirm the cachexia status of those patients, in accordance with the criteria proposed by Evans et al., (2008) [20].

The increase in ANGPTL-4 levels in plasma and tumor tissue in cancer cachexia patients was also positively correlated and associated to the levels of proinflammatory factors in the tumor and mesenteric adipose tissues. In addition, the levels of NFκB and TNF-R1 were higher in the mesenteric adipose tissue of cancer cachexia patients, and lower levels of FADD and NFκB in tumor tissue were also observed.

Previous studies have demonstrated that ANGPTL-4 expression is regulated by hypoxia in both endothelial cells [13] and tumor cells [21]. The studies are controversial when considering ANGPTL-4 involvement in cancer. Nakayama et al., (2011) [17] demonstrated that ANGPTL-4 produced by tumor cells modifies vascular endothelial cell-cell junctions, raises the permeability of capillaries, and stimulates the transendothelial passage of tumor cells. These effects facilitate the formation of micrometastases, suggesting that ANGPTL-4 may be one of the factors that participate in the progression of colorectal cancer. Conversely, Galaup et al., (2006) [15] xenografted Lewis lung carcinoma metastatic 3LL or melanoma B1670 in mice and showed that these cells metastasized less efficiently in mice that produced ANGPTL-4, via the inhibition of vascular activity and tumor cell motility and invasion.

El-Shal et al., (2017) [21] evaluated plasma levels and tissue expression of ANGPTL-3 and 4 in patients with hepatocellular carcinoma and chronic hepatitis and in healthy patients. Plasma concentrations of ANGPTL-3 were higher in patients with hepatocellular carcinoma and those with chronic hepatitis as compared with patients in the control group. However, the expression of ANGPTL-4 was higher in patients with hepatocellular carcinoma as compared with those in the other two groups. Contrary to these observations, we found an increase in the concentration of ANGPTL-4 only in cancer-cachexia patients as compared to the other two groups. Taken together, it is reasonable to suggest that ANGPTL-4 secretion in cancer depends on the type of tumor cell and may be related to the development of cachexia, depending of tissues levels and plasma concentration.

ANGPTL-4 secretion is stimulated by proinflammatory conditions [22]. In fact, our results show a significant positive correlation between ANGPTL-4 tumor levels and those of IL-1 β, TNF-α, and NFκB, and a tendency toward a positive correlation of MCP-1 tumor levels (p < 0.06) in cancer cachexia patients. Also, ANGPT-4 was positive associated was with IL-1β and MCP-1 in CC group. However, these results were not observed in cancer patients with a stable weight. Taking into account an increased TNF-R1 and NFκB protein content and association of ANGPT-4 level with IL-1β in mesenteric adipose tissue in cancer cachexia patients in comparison to cancer patients with a stable weight, and the previously reported high proinflammatory condition in the peritumoral adipose tissue of cancer cachexia patients relative to cancer patients with a stable weight [23], there is an indication that the inflammatory pathways are more active in cancer cachexia patients than in cancer patients with a stable weight and that the higher levels of ANGPTL-4 were involved with the increase of the proinflammatory factors in cancer cachexia patients.

Cinkajzlová et al., (2018) [24] evaluated the concentration of ANGPTL-4 in a different clinical situation with obese, diabetic patients with anorexia nervosa undergoing bariatric surgery with acute fasting and parenteral nutrition. It was demonstrated that fasting promotes increased plasma concentration of ANGPTL-4. This led us to believe that the proinflammatory condition combined with decreased food consumption and anorexia, which may be present in patients with cancer cachexia, may be responsible for the increased levels of ANGPTL-4 in these patients as compared to the stable weight and control groups.

ANGPTL-4 acts on adipose tissue catabolism by decreasing lipoprotein lipase activity. van der Kolk et al., (2018) [25] reported that after weight loss, plasma ANGPTL-4 was positively associated with fasting, postprandial adipose tissue NEFA and glycerol release, reflecting adipose tissue lipolysis. Fouladiun et al., (2005) [9] reported that the loss of adipose tissue mass occurs earlier and faster than the loss of muscle mass, and that this reduction in adipose tissue in cancer patients can be explained by hormonal changes and inflammatory process.

Several metabolic alterations occur in adipose tissue in cancer cachexia patients: an increase in lipolysis [26]; a decrease in lipoprotein lipase activity [27]; and *de novo* lipogenesis enzymes [28]. Accordingly, we suggest that the increase in plasma and tumor ANGPTL-4 levels in cancer cachexia patients may be partially responsible for the development of pre-cachexia and the progression to refractory cachexia.

Positive associations of ANGPTL-4 with IL-10 and IL-15 and IL-15 in the tumor and the mesenteric adipose tissue were observed in WSC group. Santana Carrero et al (2019) [29] demonstrated that IL-15 produced in response to inflammatory conditions is important for the regression of tumors and contributes to an antitumor immunity in tumor microenvironment. Moreover, IL-15 appears to play a role on the preservation of body mass and fat-free mass and the reduction of this cytokine in patients with cancer is related to the development of cachexia [30].

The development of tumor is associated with a decrease of apoptosis process in the tumor cells [11]. Previously, we found an increase in FADD levels in peritumoral adipose tissue in cancer cachexia patients in comparison to cancer patients with a stable weight [23]; however, we detected a decrease in this factor in the CC group as compared to the WSC group. The role of FADD in apoptosis is controversial; some studies have reported an anti-apoptotic effect and others a pro-apoptotic action in tumor cells [11].

A recent review reported that FADD plays a role in tumorogenesis and is related to poor prognosis in certain cancer patient, and that FADD is a key signaling molecule involved in most signalosome such as inflammasome [31]. Also, a cohort study demonstrated that the release of FADD from tumor biopsies was positively correlated with cancer stage and metastasis process [32]. Therefore, the decrease in FADD in tumor of CC patient could be related to FADD release, which could contribute to the inflammatory process involved on cancer cachexia development.

Studies have demonstrated the role of ANGPTL-4 in promoting increased tumor cell progression and aggressiveness [33, 34]. Li et al., (2015) [33] showed that tumor tissues from patients with colorectal cancer led to an overexpression of ANGPTL-4 as compared to healthy adjacent tissue. In addition, it was demonstrated that HCPT116 colorectal cancer cells overexpressing ANGPTL-4 have a lower rate of apoptosis due to a decrease in the mRNA and protein levels of Bax (a pro-apoptotic protein) and an increase in Bcl-xl levels (inhibitor of apoptosis).

Kim et al., (2011) [35] demonstrated that ANGPTL-4 promotes the proliferation of tumor cells in *in vivo* and *in vitro *models; however, changes in the apoptotic pathway were not observed. Additionally, it was shown that c-ANGPTL-4 induces the expression of STAT-1 at both the protein and mRNA levels. However, we observed no changes in the expression of STAT-1, ERK, or Src proteins in any of the tissues analyzed; therefore, we suggest that the proliferation of tumor cells occurs through a pathway independent of STAT-1. Nevertheless, an increase in the expression of ANGPTL-4 was seen only in the tumor tissue of patients in the cancer cachexia group; this increase was not observed in the adipose tissues studied.

Some of the limitations of the present study are: subjects´ previous self-reported body weight; limited sample size due to difficulty in obtaining samples from tumor tissue and subcutaneous and mesenteric adipose tissue during surgery which could result in a type 2 statistical error in the null results; not all analyses were performed for all subjects enrolled due to technical issues. Nevertheless, to the best of our knowledge, this is the first study highlighting an increase in ANGPTL-4 plasma and tumor concentrations in colorectal cancer cachexia patients relative to cancer patients with a stable weight, suggesting that ANGPTL-4 may play a role in the modulation of colorectal cancer cachexia.

## METHODS

The present work assembled a total of 102 patients from the University Hospital. Patients received detailed information regarding the research beforehand and signed the informed consent. The inclusion criteria were that patients did not receive anticancer therapy or continuous anti-inflammatory treatment. The exclusion criteria were liver failure, renal failure, HIV infected patients, inflammatory diseases of the bowel, and autoimmune disorders. Subjects general data are shown in Table 1. The fully informed written consent signature was obtained from every patient after a detailed explanation of the study. The study was approved by the Ethics Committee of the Institute of Biomedical Sciences, by the Human Ethics Committee of the University of São Paulo Hospital and by the Ethics Committee of the University Federal of São Paulo (CEP-ICB/USP 995.381, CEP-HU/USP1.027.703, CEP- UNIFESP 972.914, CAAE 39558214.0.0000.5505), and was conducted in accordance to the principles of the Declaration of Helsinki (2013). Prior to surgery, the patients completed the European Organization for the Research and Treatment of Cancer Quality of Life Questionnaire (EORTC-QLQ-C30), where fatigue and anorexia assessment were made possible. Patients were then divided into three groups: control, patients without cancer submitted to a surgical procedure (C group, n = 37); cancer patients with a stable weight, without great changes in body weight during the previous year and a BMI < 25 kg/m² (WSC group, n = 23); and those with cancer cachexia, with a reported weight loss > 5% in the previous 6 months or any degree of weight loss > 2% in the previous 6 months plus a BMI < 20 kg/m2 and the presence of systemic inflammation, decreased serum hemoglobin, and fatigue and/or anorexia (CC group, n = 42), following the international consensus guidelines [20]. Subjects from both treatment groups presented the intestine as the primary tumor location, which was then resected in all cancer patients. Along with mesenteric and subcutaneous adipose tissue, tumor was dissected and stored at -80°C until analysis.

### Clinical parameter assessment

Previous to surgery, the subjects height and weight were measured and a blood sample was collected, for quantitation of the plasma concentrations of C-reactive protein (CRP), albumin, and hemoglobin plasma. The plasma samples were immediately frozen at -80°C until analysis. TNM tumor staging was determined postoperatively according to the guidelines of the Union for International Cancer Control.

### Analysis of protein content and ANGPTL-4 expression

Protein extraction from tumor, mesenteric and subcutaneous adipose tissues, and multiplex analysis were performed as described previously by Neto et al., 2018 [23].

ANGPTL-4 was assessed by DuoSet ELISA Development kits (DY3485) from R&D Systems (Abingdon, UK), using one lot of detection antibodies XPQ0413021. The samples were read in Epoch™ Microplate Spectrophotometer, Biotek®. The results are expressed as pg/mg protein for tissues and as ng/mL for plasma. The intra- and inter-assay variability of the ANGPTL-4 kit were 6.75–11.37%, respectively.

### Statistical analysis

The data are expressed as the mean ± standard error for variables with normal distribution, or the median [interquartile range] for those not normally distributed. All samples were tested for Gaussian distribution using either the D’Agostino–Pearson omnibus test or the Shapiro–Wilk test. A Student’s t-test or the Mann–Whitney test with multiple comparisons was employed for parametric and non-parametric data, respectively. The significance level was set at p ≤ 0.05. Pearson’s or Spearman’s correlation coefficients were calculated to assess the relationships between variables. The Simple Linear Regression test was employed for detect the association of ANGPTL-4 and others factors. GraphPad Prism 5.0 was used for the analysis.

## CONCLUSIONS

In conclusion, we detected increased levels of ANGPTL-4 in both plasma and tumor tissue from patients with cancer cachexia, and a positive correlation and association were found between ANGPTL-4 and proinflammatory factors such as IL-1β content in tumor and mesenteric adipose tissue and, suggesting that the intensification of the proinflammatory environment could contribute to cancer cachexia development. Similarly, these results suggest that the increase in ANGPTL-4 and decrease in FADD in the tumor may be associated to advance of cachexia in cancer patients. Therefore in weight stable patient the ANGPTL-4 levels were lower in plasma and tumor in comparison to CC group, and increased ANGPTL-4 levels may contribute for a proinflammatory environment on cancer cachexia patients.

## Abbreviations

ANGPTL-4: Angiopoietin-like 4
C: control patients
WSC: cancer patients with a stable weight
CC: cancer-cachexia patients
TNF: tumor necrosis factor
IL: interleukin
TLR: toll-like receptor
IFN-γ: interferon gamma
EORTC-QLQ-C30: European Organization for the Research and Treatment of Cancer Quality of Life Questionnaire
BMI: body mass index
CPR: C- reactive protein
MCP-1: Monocyte Chemoattractant protein-1.
